# Measuring Lead Effects: Blood and Bone Together Are Better

**Published:** 2004-08

**Authors:** Julian Josephson

Many studies have reported impaired renal function and kidney disease at high levels of lead exposure, as estimated mainly through concentrations of serum creatinine (SCr) and rates of creatinine clearance from the body. However, lower-level lead exposure has not been correlated with renal effects as conclusively, perhaps because blood lead reflects relatively recent exposure, and therefore is not an adequate measure of total body burden. This month, Shirng-Wern Tsaih of the Harvard School of Public Health and her colleagues report that blood lead levels alone may not be enough to determine whether kidney effects are occurring at low exposure; lead levels in bone also need to be determined **[*EHP* 112:1178–1182]**. The Tsaih study is among the first to assess the relationship between low-level bone and blood lead levels and measures of kidney function in a general population sample.

In contrast with blood lead, bone lead makes up more than 95% of the adult body burden. The lead in more compacted cortical bones, such as the tibia, is less available for mobilization, because this type of bone is less prone to turnover than spongier trabecular bones, such as the patella. Yet, as people age, bone loss often does take place, so lead that has long been held in bone is released to soft tissue and can find its way to the kidneys. Thus, bone lead may be a better marker for studying the chronic toxicity of accumulated exposure and lead burden.

The Tsaih study examined data from a cohort of middle-aged and elderly Boston men with no known heavy exposure to lead. Participants were from the Normative Aging Study, a federal study of aging begun in 1961. A blood sample for lead analysis had been collected every 3–5 years since July 1988; bone lead measurements began in August 1991, when a subset of participants were recruited for a substudy in which bone lead was measured by K X-ray fluorescence.

Tsaih and colleagues examined data for 448 men who had a baseline bone lead measurement between 1991 and 1995, and follow-up measurements of SCr 4–8 years later. They examined blood and bone lead concentrations and their correlation with kidney function, taking into account the known nephrotoxic effects of diabetes mellitus and hypertension, which had been diagnosed in 5.8% and 25.7% of the men, respectively, at the time of baseline measurement. Bone lead was measured in the tibia and patella.

Tibia lead was observed to be associated with increases in SCr levels in follow-up participants with diabetes. The findings suggest that long-term low-level lead accumulation, estimated by tibia lead, is associated with an increased risk of reduced renal function. This is especially true for diabetics and hypertensives, who already are at risk for kidney impairment because of their disease. In addition, blood lead and tibia lead appeared to be associated with elevated SCr levels and chronic kidney disease among hypertensives. There was no statistical evidence of patella lead being associated with change in renal function, suggesting that chronic absorption of lead is a risk factor for impaired renal function.

The study, however, has some limitations. Although SCr is widely used in medicine to measure overall renal function, it provides only a rough estimate of the kidney’s filtration capacity. For instance, increases in SCr definitively show impairment only when kidney function has been reduced by about 50%. Thus, the researchers had great difficulty in detecting more modest effects of lead. In addition, the alternative hypothesis that elevated blood or bone lead levels actually result from impaired kidney function cannot be ruled out.

It has not yet been determined whether lead affects blood pressure indirectly through changes in kidney function, or via more direct effects on the circulatory system or neurological blood pressure control. The researchers also know of no studies to date that analyze the potential for diabetes to modify the relationship between lead exposure and renal function. Given that many adults have a history of environmental or occupational lead exposure and the incidence of both type 2 diabetes and hypertension, studies of such interactions, if confirmed, could be of significant public health value.

## Figures and Tables

**Figure:**
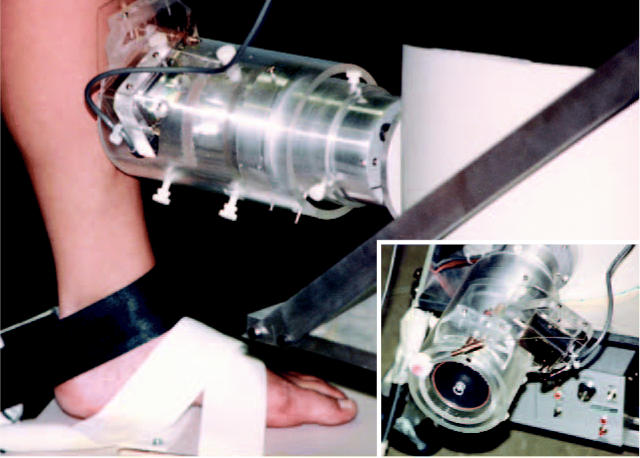
**A step in the right direction.** Correlating bone lead measurements (obtained through K X-ray fluorescence) with blood lead data yields insights into adverse renal effects.

